# Sigh-induced changes of breathing pattern in preterm infants

**DOI:** 10.14814/phy2.12613

**Published:** 2015-11-12

**Authors:** Kerstin Jost, Philipp Latzin, Sotirios Fouzas, Elena Proietti, Edgar W Delgado-Eckert, Urs Frey, Sven M Schulzke

**Affiliations:** 1 Department of Neonatology, University of Basel Children’s Hospital (UKBB)Basel, Switzerland; 2 Faculty of Medicine, Biomedical Engineering, University of BaselBasel, Switzerland; 3 University of Basel Children’s Hospital (UKBB)Basel, Switzerland; 4 Pediatric Respiratory Unit, University Hospital of PatrasRio, Greece; 5 Computational Physiology and Biostatistics, University of Basel Children’s Hospital (UKBB)Basel, Switzerland

**Keywords:** Bronchopulmonary dysplasia, control of breathing, preterm infant, sigh, variability

## Abstract

Sighs are thought to play an important role in control of breathing. It is unclear how sighs are triggered, and whether preterm birth and lung disease influence breathing pattern prior to and after a sigh in infants. To assess whether frequency, morphology, size, and short-term variability in tidal volume (*V*_T_) before, during, and after a sigh are influenced by gestational age at birth and lung disease (bronchopulmonary dysplasia, BPD) in former preterm infants and healthy term controls measured at equivalent postconceptional age (PCA). We performed tidal breathing measurements in 143 infants during quiet natural sleep at a mean (SD) PCA of 44.8 (1.3) weeks. A total of 233 sighs were analyzed using multilevel, multivariable regression. Sigh frequency in preterm infants increased with the degree of prematurity and severity of BPD, but was not different from that of term controls when normalized to respiratory rate. After a sigh, *V*_T_ decreased remarkably in all infants (paired *t*-test: *P* < 0.001). There was no major effect of prematurity or BPD on various indices of sigh morphology and changes in *V*_T_ prior to or after a sigh. Short-term variability in *V*_T_ modestly increased with maturity at birth and infants with BPD showed an earlier return to baseline variability in *V*_T_ following a sigh. In early infancy, sigh-induced changes in breathing pattern are moderately influenced by prematurity and BPD in preterm infants. The major determinants of sigh-related breathing pattern in these infants remain to be investigated, ideally using a longitudinal study design.

## Introduction

Sighs, that is, large tidal breaths at least double the average tidal volume (*V*_T_) of the preceding breaths, have been associated with various physiological and pathophysiological mechanisms. Sighs play an important role in the plastoelastic stretching of lung tissue and breathing muscles, which may result in improvement of lung compliance, reduction in airway resistance and recruitment of lung volume (Davis and Moscato 1994). Additionally, the effect of sighs depends on subject characteristics such as age: Sighs can lead to hypoventilation and apnea in infants, but might induce higher minute ventilation in adults. It is, however, unknown whether maturation of respiratory control systems or biomechanical lung development is the primary cause of these results (Qureshi et al. 2009).

Baldwin et al. described decreased short-range breath-to-breath memory prior to and increased variability in *V*_T_, and minute ventilation immediately following a sigh in healthy term infants, measured 5 weeks after their expected date of delivery during quiet sleep (Baldwin et al. 2004). These findings were interpreted as additional evidence that the ability to sigh may play an important role in control of breathing. The authors hypothesized that the reaction to a sigh might differ between term and preterm infants due to differences in both control of breathing and lung function, and that it could be a potential marker for respiratory sequelae in preterm infants. The role of sighs in preterm infants is poorly understood, particularly in those who have bronchopulmonary dysplasia (BPD) (Qureshi et al. 2009); BPD is a chronic, developmental lung disease characterized by altered breathing pattern, poor lung function, and impaired lung growth, affecting at least 12,000 preterm infants in the United States each year (Jobe and Bancalari 2001; Baldwin et al. 2006; Hulskamp et al. 2009; Latzin et al. 2009b; Van Marter 2009).

We thus hypothesized that the frequency, morphology, and short-term variability in tidal breathing differ between healthy term infants and preterm infants with BPD when measured at the same corrected age. We further hypothesized that differences in those outcomes are mainly influenced by the degree of prematurity at birth and severity of BPD. Thus, we aimed to assess whether frequency, morphology, and short-term variability of *V*_T_ before, during, and after a sigh are influenced by gestational age (GA) at birth, and presence and severity of BPD in former preterm and healthy term control infants.

## Methods

### Study design

This is a retrospective analysis of data obtained from a prospective birth cohort study conducted in Bern, Switzerland (BILD cohort study) (Latzin et al. 2009a). Infants had participated in tidal breathing measurements according to European Respiratory Society (ERS) standards (Bates et al. 2000) from September 2002 to December 2009. The study was approved by the Bernese Ethics Committee and written informed consent was obtained for each subject prior to the measurement.

### Patients

**Figure 1:**
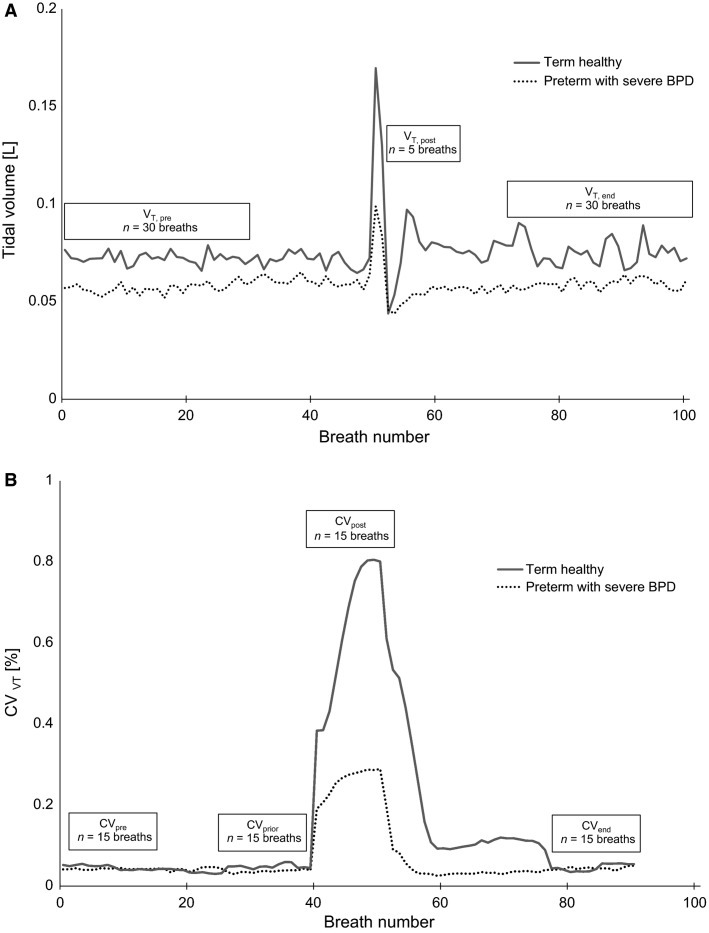
Representative tidal breathing traces of *V*_T_ (A) and coefficient of variation in *V*_T_ (CV_VT_) (B) for an infant with severe bronchopulmonary dysplasia (BPD) and a term healthy control infant. *V*_T,pre_, mean tidal volume over the first 30 breaths of the measurement; *V*_T,post_, mean tidal volume over the first five breaths after the sigh; *V*_T,end_, mean tidal volume over the last 30 breaths of the measurement; CV_pre_, CV_post_, and CV_end_, coefficient of variation in *V*_T,pre_, *V*_T,post_, and *V*_T,end_, respectively; CV_prior_, coefficient ofvariation in *V*_T_ over 15 breaths preceding a sigh.

We studied former preterm infants (*n* = 57) born at <37 weeks GA and healthy term control infants (*n* = 86). Preterm infants were assessed for presence of BPD based on their duration of supplemental oxygen requirement at 36 weeks GA. Mild, moderate, and severe BPD was defined based on the National Institutes of Child Health Consensus definition (Jobe and Bancalari 2001). Figure1 shows a flow chart of patients through the phases of the study.

### Measurements

Detailed measurement set up has been published previously (Fuchs et al. 2012). Briefly, measurements were conducted at a mean (range) postconceptional age (PCA) of 44.8 (41.7–51.9) weeks in infants without any respiratory infections. Tidal breathing measurements lasted up to 10 min and were conducted following international guidelines for lung function testing in infancy (Bates et al. 2000). Measurements were conducted with the infant in supine position, during behaviorally defined, quiet unsedated sleep (Prechtl 1974). A face mask was placed on the infant’s mouth and nose during regular tidal breathing (size 1, Homedica, Cham, Switzerland). The mask was connected to an infrared CO_2_ analyzer and an ultrasonic flowmeter (Spiroson Exhalyzer D, EcoMedics AG, Duernten, Switzerland) as described previously (Latzin et al. 2009b). A bypass flow of 14 L/min was applied. End-tidal CO_2_ was monitored for the entire measurement period and did not increase. Signals were 12-bit analog-to-digital converted and sampled at a frequency of 200 Hz using a commercially available data acquisition and analysis package (WBreath version 3.7.6.0, Firmware v3.06, NDD Medizintechnik AG, Zürich, Switzerland).

### Data processing

After BTPS correction and correction for flow offset, we visually examined all tidal breathing measurements for sighs. We aimed to analyze a minimum of 50 baseline breaths before and after the sigh, respectively. Breath traces were processed in the statistical software R (R Core Team [2013]. R: A language and environment for statistical computing. R Foundation for Statistical Computing, Vienna, Austria.), and sighs were automatically identified by software script according to the following criteria: (1) Sigh *V*_T_ > 2 standard deviations (SD) of average *V*_T_ of preceding breaths (Thach and Taeusch 1976; Davis and Moscato 1994); (2) Minimal distance of 10 breaths to preceding or following sigh; (3) Availability of at least 5 breaths before and 10 breaths after each sigh. If those criteria were not fulfilled, measurements were excluded from analysis.

### Data analysis

The following parameters were then analyzed for all measurements containing at least one sigh that met inclusion criteria:

#### Sigh frequency

All measurements lasting at least 10 min were used for the analysis of sigh frequency. Respiratory rate (mean over 10 min), number of sighs within 10 min, and extrapolated value of sighs expected in 1000 breaths were defined.

#### Sigh morphology

Maximal inspiration (*V*_I,max_) and maximal expiration (*V*_E,max_) during a sigh (absolute values and values corrected for *V*_T_ at baseline), and the ratio of *V*_E,max_/*V*_I,max_ were calculated to describe the morphology of a sigh.

#### Changes in *V*_T_

The first 30 breaths of the tidal breathing measurement were used as baseline before the sigh (*V*_T,pre_), the last 30 breaths as baseline after the sigh (*V*_T,end_). The five breaths just after the sigh were defined as immediate postsigh period (*V*_T,post_). We then calculated the difference in mean *V*_T,pre_ − *V*_T,post_ as a measure of changes in *V*_T_ upon a sigh (*V*_T,diff_). We further calculated the number of breaths that deviated from *V*_T,pre_ in excess of 2 SD within the periods 15 breaths before and 15 breaths after a sigh, and discriminated between the number of high (*V*_T,high_) and low (*V*_T,low_) values.

#### Short-term variability in *V*_T_

Short-term variability in *V*_T_ was determined using a moving window algorithm, in which the coefficient of variation (CV) of *V*_T_ (CV_VT_) was obtained from windows of 11 breaths. The window was shifted by one breath four times in four predefined regions of the tidal breathing measurement (baseline at the beginning, just prior to the sigh, immediately after a sigh, baseline at the end of the measurement). This approach resulted in four consecutive windows per region. Baseline CV_VT_ was calculated at the beginning (CV_pre_) and at the end (CV_end_) of a measurement. Also, CV_VT_ was calculated just prior to the sigh (CV_prior_) and immediately after a sigh (CV_post_). CV_diff_ (CV_post_ − CV_pre_) describes the change from baseline to postsigh period. We further calculated changes in CV_VT_ (CV_post_slope_) by subtracting the value of the second moving window just after the sigh from the first one just after the sigh, normalized for individual CV_pre_. Sample tidal breathing traces including a graphical overview on outcomes related to changes in *V*_T_ and variability in *V*_T_ are displayed in Figure1A and B, respectively.

### Statistical analysis

Our main outcome parameters, as described above, were number of sighs (sigh frequency); *V*_I,max_ and *V*_E,max_ (sigh morphology); size of tidal volume before and after a sigh (changes in *V*_T_), and coefficient of variation in *V*_T_ (Short-term variability in *V*_T_).

We performed linear regression analyses to assess associations between sigh characteristics (frequency, morphology, changes in *V*_T_, variability in *V*_T_) and potential predicting factors. Considered predictors included degree of prematurity (GA), BPD (expressed as number of days with supplemental oxygen, i.e., as a continuous variable), body size at test (weight), intrauterine growth restriction (birth weight *z*-score), gender, and maternal smoking during pregnancy. We used multilevel modeling to allow clustering on the individual level given that some measurements incorporated more than one sigh. Model building included exploring associations of all considered predictors with sigh characteristics in univariable regression analysis where *P* < 0.1 was considered to indicate potential relevance of a predictor. We then built multilevel, multivariable linear regression models for each outcome and did stepwise backward elimination of predictors that were not significantly associated with the outcome (*P* < 0.05 considered statistically significant). Lastly, we defined a best model depending on the coefficient of determination of the model (*R*^2^). Statistical analysis was done using Stata software (StataCorp. 2009. Stata Statistical Software: Release 11. College Station, TX: StataCorp LP).

## Results

**Figure 2:**
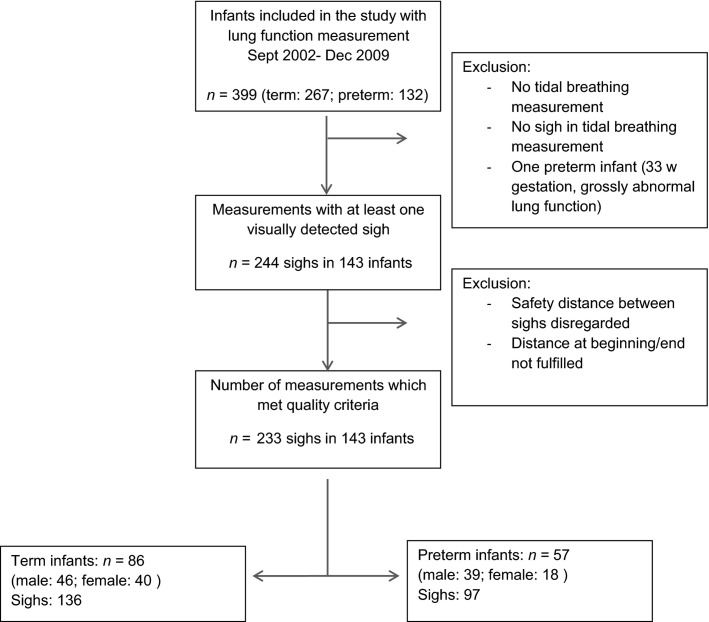
Flow of patients through the phases of the study.

We screened data from 399 infants. Out of these, 143 (35.8%) showed at least one sigh during their tidal breathing measurement and were included in this study. A total of 233 sighs met inclusion criteria and were used for further analysis (Fig.2). Demographic data and tidal breathing outcomes are summarized in Tables1 and 2, respectively. Multilevel models are detailed in Table3.

**Table 1:** Demographic characteristics of study participants

	Term healthy (*n* = 86)	Preterm without BPD (*n* = 35)	Preterm with BPD (*n* = 22)
Sex, male (% male)	46 (53%)	23 (66%)	16 (73%)
Gestational age (w)	39.4 (37.0, 41.9)	30.6 (24.1, 35.6)	27.4 (24.3, 31.6)
Birth weight (kg)	3.3 (2.5, 4.9)	1.5 (0.5, 3.0)	1.0 (0.4, 2.6)
Birth weight *z*-score	−0.12 (−2.07, 2.75)	−0.60 (−3.58, 2.29)	−0.48 (−4.03, 3.46)
Study weight (kg)	4.3 (3.2, 6.4)	4.3 (2.7, 6.8)	3.4 (2.6, 5.6)
Postconceptional age (w)	44.3 (41.7, 48.0)	44.9 (43.4, 47.0)	44.7 (43.3, 51.9)
Infants with sighs/measured infants (%)	86/223 (39)	35/94 (37)	22/82 (27)
Number of observed sighs	136	52	45

Values are described as mean (range). BPD, bronchopulmonary dysplasia.

**Table 2:** Tidal breathing outcomes

	Term healthy *n* = 86	Preterm without BPD *n* = 35	Preterm with BPD *n* = 22
Respiratory rate	42.60 (7.84)	48.32 (12.45)	49.92 (13.01)
Sighs/10 min	1.52 (0.65)	1.41 (0.67)	2.00 (1.08)
Sighs/1000 breaths	3.67 (1.69)	3.16 (1.77)	4.28 (2.40)
*V*_I,max_/*V*_T,pre_	3.07 (0.99)	2.92 (0.65)	2.76 (0.63)
*V*_E,max_/*V*_T,pre_	2.32 (0.68)	2.37 (0.62)	2.50 (0.45)
*V*_E,max_/*V*_I,max_	0.79 (0.18)	0.84 (0.21)	0.93 (0.16)
*V*_T,pre_ (mL)	32.95 (5.67)	32.17 (7.60)	27.31 (4.96)
*V*_T,post_ (mL)	23.18 (7.74)	23.04 (6.58)	20.53 (6.25)
*V*_T,diff_ (mL)	9.76 (7.28)	9.01 (6.92)	6.78 (4.93)
VT_high_ after sigh (breaths)	4.69 (4.84)	5.02 (5.36)	3.39 (4.55)
*V*_T,end_ (mL)	32.71 (5.61)	30.71 (7.37)	26.48 (4.96)
CV_pre_	0.08 (0.06)	0.06 (0.03)	0.07 (0.06)
CV_prior_	0.09 (0.10)	0.08 (0.07)	0.08 (0.06)
CV_post_	0.37 (0.16)	0.32 (0.12)	0.27 (0.15)
CV_diff_	0.30 (0.16)	0.26 (0.12)	0.20 (0.16)
CV_post_slope_	0.96 (0.92)	1.58 (1.80)	2.58 (2.26)
CV_end_	0.10 (0.07)	0.09 (0.08)	0.07 (0.03)

Values are given as mean (SD).

**Table 3:** Results of multilevel, multivariable regression analyses on sigh frequency, sigh morphology, tidal volume and variability in tidal volume

	Multivariable models			*R* ^2^
	Coefficient	CI 95%	*P*-value	
Respiratory rate				
Gestational age (w)	−0.5433	−0.8995, −0.18722	0.003	0.15
Birth weight *z*-score	−2.4207	−4.2435, −0.5980	0.010	
Sigh frequency				
Sighs/10 min				
BPD	0.0047	−0.0002, 0.0096	0.059	0.03
Sigh morphology				
*V*_E,max_/*V*_T,pre_				
BPD	0.0036	0.0004, 0.0068	0.028	0.06
Maternal smoking	−0.4936	−0.8383, −0.1489	0.005	
*V*_E,max_/*V*_I,max_				
BPD	0.0016	0.0008, 0.0023	<0.001	0.09
Maternal smoking	−0.0791	−0.1606, 0.0023	0.057	
Changes in *V*_T_				
*V*_T,pre_				
Gestational age (w)	0.0003	0.0001, 0.0004	0.001	0.40
Weight (kg)	0.0048	0.0037, 0.0061	<0.001	
Sex, male	0.0016	−0.0001, 0.0034	0.058	
*V*_T,post_				
Weight (kg)	0.0034	0.0018, 0.0050	<0.001	0.12
Sex, male	0.0023	0.0002, 0.0045	0.036	
*V*_T,diff_				
BPD	−0.0000	−0.0001, −0.0000	0.024	0.04
VT_high_ after sigh (breaths)				
BPD	−0.0297	−0.0515, −0.0079	0.008	0.03
*V*_T,end_				
Gestational age (w)	0.0003	0.0017, 0.0005	<0.001	0.40
Weight (kg)	0.0045	0.0032, 0.0057	<0.001	
Sex, male	0.0020	0.0002, 0.0037	0.027	
Short-term variability in *V*_T_				
CV_pre_				
Gestational age (w)	0.0008	−0.0004, 0.0020	0.190	0.05
Sex, male	−0.0930	−0.1521, −0.0338	0.002	
CV_post_				
Gestational age (w)	0.0073	0.0039, 0.0107	<0.001	0.16
Sex, male	−0.0394	−0.0786, −0.0002	0.043	
Maternal smoking	−0.0692	−0.1350, −0.0035	0.041	
CV_diff_				
Gestational age (w)	0.0055	0.0013, 0.0096	0.009	0.07
Maternal smoking	−0.0940	−0.1733, −0.0146	0.020	
CV_post_slope_				
BPD	0.0188	0.0122, 0.0255	<0.001	0.15
CV_end_				
Gestational age (w)	0.00102	−0.0010, 0.0031	0.025	0.16
Sex, male	−0.0323	−0.0528, −0.0118	0.003	
Birth weight *z*-score	0.0172	0.0078, 0.0265	<0.001	

Bronchopulmonary dysplasia (BPD) was expressed as the number of days on supplemental oxygen. Respiratory rate was averaged over the duration of the measurement. *V*_I,max_/*V*_T,pre_, maximal inspiratory volume during the sigh normalized to mean tidal volume at the beginning of the measurement; *V*_E,max_/*V*_T,pre_, maximal expiratory volume during the sigh normalized to mean tidal volume at the beginning of the measurement; *V*_E,max_/*V*_I,max_, ratio of maximal expiratory volume during the sigh/maximal inspiratory volume during the sigh; *V*_T,pre_, mean tidal volume over the first 30 breaths of the measurement; *V*_T,post_, mean tidal volume over the first five breaths after the sigh; *V*_T,diff_, difference between *V*_T,pre_ and *V*_T,post_; *V*_T,high_, number of breaths after a sigh that exceeded 2 SD of *V*_T,pre_; *V*_T,end_, mean tidal volume over the last 30 breaths of the measurement; CV_pre_, coefficient of variation in *V*_T,pre_; CV_prior_, coefficient of variation in *V*_T,prior_; CV_post_, coefficient of variation in *V*_T,post_; CV_diff_, difference between CV_pre_ and CV_post_ as an estimate of change in variability in tidal breathing upon a sigh; CV_post_slope_, difference between CV_VT_ of first window after the sigh and second window after the sigh normalized to individual baseline CV_VT_; CV_end_, coefficient of variation in *V*_T_.

### Sigh frequency

Respiratory rate was positively associated with BPD (*P* = 0.008) and negatively associated with GA (*P* = 0.001), body weight (*P* = 0.076), and birth weight *z*-score (*P* = 0.004). Eighty-six out of 223 term infants (39%), 35 out of 87 preterm infants without BPD (40%), and 22 out of 89 preterm infants with BPD (25%) had at least one sigh during their tidal breathing measurement. The number of sighs over a 10 min measurement period was associated with BPD (*P* = 0.059), but not GA (*P* = 0.12) (Table3). On normalizing sigh frequency to sighs per 1000 breaths, no significant associations between sigh frequency and GA, BPD or any other predictor variable were found.

### Sigh morphology

In univariable analyses, maximal inspiratory volume of the sigh (*V*_I,max_) was not associated with any predictor when corrected for baseline *V*_T_. In contrast, maximal expiratory volume of the sigh (*V*_E,max_) was positively associated with BPD (*P* = 0.038) and negatively with maternal smoking (*P* = 0.006) after correcting for *V*_T_ at baseline. Additionally, the ratio of *V*_E,max_/*V*_I,max_ was negatively associated with GA (*P* < 0.001) and maternal smoking (*P* = 0.078), and positively associated with BPD (*P* < 0.001). In multivariable analysis, *V*_E,max_/*V*_I,max_ remained weakly associated with BPD after adjusting for maternal smoking (*R*^2^ = 0.09) (Table3).

### Changes in *V*_T_

In univariable analyses, both *V*_T,pre_ and *V*_T,end_ were positively associated with GA, body weight, and male sex, but negatively associated with BPD. In contrast, *V*_T,post_ was neither associated with GA nor with BPD. The only significant predictors of *V*_T,post_ were body weight (*P* < 0.001) and sex (*P* = 0.007). *V*_T,diff_ was positively associated with GA (*P* = 0.023), body weight (*P* = 0.022), and birth weight *z*-score (*P* = 0.062), and negatively associated with BPD (*P* = 0.024). *V*_T,post_ was smaller than *V*_T,pre_ (paired *t*-test, *P* < 0.001). *V*_T,high_ after the sigh was negatively associated with BPD (*P* = 0.028). There were no associations between major deviation from *V*_T,pre_ (*V*_T,high_, *V*_T,low_) and GA or BPD.

Multivariable modeling established a positive association of both V_T,pre_ (*R*^2^ = 0.40) and *V*_T,end_ (*R*^2^ = 0.40) with GA, but not BPD after adjusting for body weight and sex (Table3). We found no multivariable model for *V*_T,diff_.

### Short-term variability in *V*_T_

In univariable analyses, CV_pre_ showed a weak positive association with GA (*P* = 0.045), but not BPD and a negative association with male sex (*P* < 0.001). CV_prior_ was not associated with any tested predictor. CV_post_ was positively associated with GA (*P* < 0.001) and negatively associated with BPD (*P* < 0.001), male sex (*P* = 0.043), and maternal smoking in pregnancy (*P* = 0.085). CV_end_ showed a positive association with GA (*P* = 0.041) and birth weight *z*-score (*P* < 0.001), and a negative association with BPD (*P* = 0.043) and male sex (*P* = 0.003). CV_diff_ as a marker of changes in CV of *V*_T_ from baseline to immediately after a sigh was positively associated with GA (*P* = 0.010), and negatively associated with BPD (*P* = 0.015) and maternal smoking (*P* = 0.022). CV_post_slope_ as a marker of how fast variability in *V*_T_ after a sigh returns down to baseline, was negatively associated with GA (*P* < 0.001) and positively associated with BPD (*P* < 0.001; *R*^2^ = 0.15).

**Figure 3:**
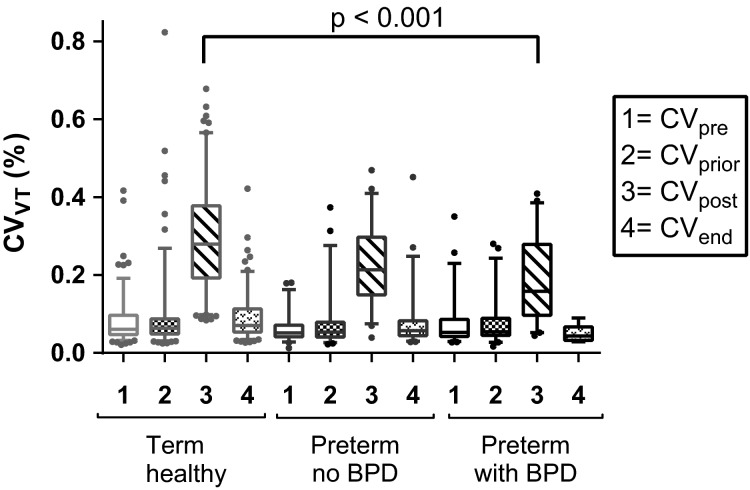
Short-term variation in tidal volume (CV_VT_) for different subgroups (Term Healthy; Preterm infants without BPD at 36 weeks GA; Preterm infants with BPD at 36 weeks GA). CV_VT_ was measured using moving window technique at the start of the measurement (CV_pre_), prior to the sigh (CV_prior_), just after a sigh (CV_post_), and at the end of a measurement (CV_end_).

Multivariable analysis (Table3) established a positive association of CV_post_ with GA, but not BPD after adjusting for sex and maternal smoking (*R*^2^ = 0.16). Similarly, CV_end_ was significantly associated with GA, but not BPD after adjusting for sex and birth weight *z*-score (*R*^2^ = 0.16). CV_diff_ was associated with GA after adjusting for maternal smoking in pregnancy (*R*^2^ = 0.07). Figure3 shows CV_pre_, CV_prior_, CV_post_, and CV_end_ for the subgroups of healthy term infants, preterm infants without BPD, and preterm infants with BPD.

## Discussion

We found that sigh-induced changes in breathing pattern differ modestly between stable preterm infants with and without BPD and term healthy controls when measured during quiet sleep at equivalent PCA shortly after term. Sigh frequency in preterm infants increased with the degree of prematurity at birth and severity of BPD. Sigh frequency of preterm infants was not different from that of term healthy controls when normalized to respiratory rate. There was no major effect of prematurity or BPD on various indices of sigh morphology indicating that former preterm infants were able to recruit similar amounts of *V*_T_ during a sigh despite their known restrictive lung disease (Choukroun et al. 2013; Schmalisch et al. 2013). Changes in *V*_T_ immediately prior to or after a sigh did not substantially differ between preterm and term infants. Although infants were studied at equivalent PCA, short-term variability in *V*_T_ modestly increased with maturity at birth and infants with BPD showed an earlier return to baseline variability in *V*_T_ following a sigh.

### Comparison with previous literature

To the best of our knowledge, this is the first study examining sigh-related breathing pattern during quiet sleep in former preterm and term healthy control infants measured at equivalent PCA shortly after term corrected age. Qureshi et al. (2009) compared sigh-related tidal breathing of 10 term and 10 preterm infants (mean PCA of 33–34 weeks) within the first 3 weeks of life to that of 10 healthy adults. They found a higher frequency of sighs in infants compared to adults but no significant difference in sigh frequency, sigh morphology, and changes in *V*_T_ between preterm and term infants. Sigh frequency normalized to respiratory rate, variability in *V*_T_, and the influence of concomitant factors on outcomes was not assessed in their study. It is somewhat surprising that Qureshi et al. did not find differences in sigh frequency between preterm infants at about 33–34 weeks PCA versus term infants at 41 weeks PCA given the maturational discrepancy of over 8 weeks during a critical period of development of respiratory control (Engoren et al. 2009); many investigators believe that sighs occur more frequently in preterm infants due to their pronounced need of restoring lung volume (Brockmann et al. 2011). However, postnatal age of preterm infants in Qureshi et al. was close to that of term infants (17 ± 3 vs. 11 ± 2 days), and the occurrence of spontaneous sighs during quiet sleep in infants is indeed related to their postnatal age: Within the first weeks of life, sigh frequency drops from about 0.9 sighs/min to 0.2 sighs/min as indicated by serial pneumography in infants studied from day one of life until 7 months of age (Fleming et al. 1984). Further, differences in the definition of a sigh (≥100% above baseline *V*_T_ in our study vs. ≥50% above baseline *V*_T_ in Qureshi et al.) and experimental conditions (supine position 30 min postfeed in our study versus supine or lateral position pre- or postfeed in Qureshi et al.) might explain the discrepancy to our results as breathing pattern of preterm infants measured in left lateral and prone position differs from that obtained in supine position (Gouna et al. 2013). Our findings of a decrease in *V*_T_ and increased short-term variability in *V*_T_ after a sigh are in agreement with our earlier findings reported by Baldwin et al. who studied variability as well as short- and long-range memory of *V*_T_ in term healthy infants at 4–6 weeks postnatal age (Baldwin et al. 2004). In this previous study, we found stability in long-range memory but improved variability and short-range memory of *V*_T_ after a sigh. This study further shows that changes in *V*_T_ and the temporary gain in short-term variability in *V*_T_ following a sigh are less pronounced in former preterm infants and that infants suffering from BPD return faster to their lower baseline variability in *V*_T_ after a sigh (CV_post_slope_). This suggests that both immaturity at birth and residual lung disease accelerate an infant’s return to baseline breathing pattern after a sigh. However, the effect size of both preterm birth and BPD is small indicating that unmeasured factors substantially influence those outcomes.

### Strengths and limitations

All measurements were conducted according to American Thoracic Society/European Respiratory Society standards for infant lung function testing. Infants were studied at a comparable PCA and were assessed in unsedated quiet sleep using modern, miniaturized lung function equipment. Limitations of our study include a recruitment period of over 7 years, potentially introducing observer bias as several investigators conducted the measurements. Nevertheless, all personnel followed standard operating instructions and we did not find any trends in outcomes over time, that is, observer-dependent effects as origin of our findings are unlikely. A general methodological difficulty lies in the precise quantification of residual lung disease in preterm infants as the clinical definition of BPD is entirely based on duration, and level of oxygen supplementation and respiratory support during neonatal intensive care stay (Jobe and Bancalari 2001). This simplistic approach might lead to misclassification, however, currently there is no superior alternative diagnostic tool and it remains a valid predictor of poor outcome including death and long-term respiratory and neurological sequelae (Kugelman et al. 2007; Schmidt et al. 2003).

### Interpretation and mechanisms

We expected a higher sigh frequency in preterm versus term infants due to the particular need of preterm infants to restore lung volume, optimize compliance and resistance, and, presumably, to reset autonomic tone (Alvarez et al. 1993; Davis and Moscato 1994; Poets et al. 1997). The observation that sigh frequency normalized to respiratory rate did not differ between preterm and term infants underlines the importance of baseline breathing pattern as a marker of maturation in preterm infants: Both *V*_T,pre_ and *V*_T,end_ were significantly associated with GA at birth; further, respiratory rate in preterm infants was increased compared to term infants although all patients were measured at equivalent PCA. This is in agreement with Schmalisch et al. who found similar associations in a lung function study of 386 very low- birth weight infants measured at 48–52 weeks of PCA (Schmalisch et al. 2013). Arguably, sigh frequency in former preterm infants during quiet sleep is a function of respiratory rate, which in turn reflects biological maturity of the respiratory system.

The specific mechanisms that trigger sighs in human infants are essentially unknown (Alvaro and Rigatto 2011). We found that contrary to baseline breathing pattern, *V*_T_, variability in *V*_T_ immediately prior to a sigh, and morphology of the sigh itself are fairly similar between preterm and term infants. We can only speculate on the reasons for such “uniformity of sigh-breathing”; based on our findings, the presigh and sigh period during quiet sleep represent epochs of respiration that are genuinely independent of maturity at birth, degree of residual lung disease, and baseline demographics such as body weight or sex. Most remarkably, inspiratory sigh volume (*V*_I,max_) of preterm infants was comparable to that of their term peers although these infants have restrictive lung disease (Thunqvist et al. 2014). However, the reaction to a sigh seems to be influenced by GA and BPD; depth and duration of change in breathing pattern after a sigh are associated with both prematurity and residual lung disease: After the sigh, preterm infants, and particularly those with BPD return faster to their lower baseline variability in *V*_T_. These observations are consistent with the hypothesis that although sighs play an important role in restoring lung volume in preterm infants (Poets et al. 1997), these infants might not tolerate prolonged deviations from baseline breathing pattern due to underlying maturational deficits. The reason(s) for this phenomenon are unclear. We speculate that the potential threat of hypoventilation after a sigh requires preterm infants, and especially those with BPD, to quickly return to baseline breathing pattern in order to avoid an epoch of periodic breathing/hypoxia under conditions of an immature respiratory feedback loop (Thach and Taeusch 1976; Bradley 2002; Khan et al. 2005; Qureshi et al. 2009). This behavior may indicate an immature respiratory pattern generator which potentially could be important for cardio–respiratory coupling and survival of infants under stress (Ramirez 2014).

We conclude that breathing pattern following a sigh is moderately influenced by the degree of prematurity and residual lung disease in preterm and term infants measured at equivalent corrected age shortly after the expected date of delivery. The precise mechanisms triggering sighs, and the major determinants of breathing pattern prior to and after a sigh in preterm infants remain to be investigated in future studies. Whether or not sigh architecture is a predictive marker of later respiratory morbidity should ideally be investigated in future longitudinal studies. Such studies should potentially include measurement of long-range memory of control of breathing given that the latter provided novel insights into breathing dynamics in term healthy infants.

## Aknowledgements

We thank Nitin Kumar for his assistance in computer programming and Karine Landgren-Hugentobler for reviewing of the manuscript.

## Conflict of Interest

None declared.
